# REACT SHOCK trial protocol and analysis plan—a multicenter randomised controlled trial comparing individualised blood pressure target versus standard blood pressure target among critically ill patients with shock

**DOI:** 10.1186/s13063-025-09142-9

**Published:** 2025-10-14

**Authors:** Rakshit Panwar, Alison Gibberd, Chris Oldmeadow, Ravindranath Tiruvoipati, Anders Åneman, Amit Kansal, REACT SHOCK investigators

**Affiliations:** 1 Intensive Care Unit, John Hunter Hospital, Newcastle, Australia; 2 School of Medicine and Public Health, University of Newcastle, Newcastle, Australia; 3 Data Sciences, Hunter Medical Research Institute, Newcastle, Australia; 4 Melbourne School of Population and Global Health, University of Melbourne, Melbourne, Australia; 5 Intensive Care Unit, Peninsula Health, Frankston, Australia; 6 Peninsula Clinical School, Monash University, Frankston, Australia; 7 Department of Epidemiology and Preventive Medicine, Monash University, Melbourne, Australia; 8 Department of Intensive Care, Liverpool Hospital, Sydney, Australia; 9 Department of Intensive Care Medicine, Ng Teng Fong General Hospital, National University Health System, Singapore, Singapore; 10 National University of Singapore, Singapore, Singapore

**Keywords:** Critical illness, Shock, Blood pressure targets, Relative hypotension, Randomised clinical trial, Mortality, Protocol, Statistics

## Abstract

**Background:**

Critically ill patients with shock receiving vasopressor or inotrope therapy in ICU are often exposed to relative hypotension, which is quantified as percentage blood pressure deficit relative to usual pre-illness blood pressure. Whether minimising such blood pressure deficit, by adjusting blood pressure targets according to patients’ pre-illness blood pressure (individualised blood pressure target strategy), can improve clinical outcomes remains unclear. Therefore, we are conducting a multicenter randomised controlled trial, the REACT SHOCK RCT, comparing individualised blood pressure targets to standard care among critically ill patients with shock.

**Methods:**

The REACT SHOCK RCT is an international, multicenter, parallel-group, randomised, standard-care controlled, clinical superiority trial that will be conducted in up to 35 ICUs in Australia, Ireland, Singapore, UK and USA. In total, 1260 patients, receiving vasopressor therapy for non-haemorrhagic shock in ICU, will be randomly assigned to individualised mean arterial blood pressure (MAP) targets (determined as an average of 2–5 recent pre-illness blood pressure readings within last 3 years, with a MAP target range of 55 to 95 mmHg) or standard care (default MAP target of 65 mmHg) in a 1:1 ratio. The REACT SHOCK RCT is anticipated to complete recruitment by 2028. The primary endpoint is all-cause 14-day mortality. Secondary endpoints are major adverse kidney events by day 14, all-cause 90-day mortality, survival time to 14 days and 90 days, and renal replacement therapy free days by day 28.

**Discussion:**

The REACT SHOCK RCT is the first international multicenter randomised clinical trial designed to ascertain whether an individualised blood pressure target strategy is superior to standard care for critically ill patients with shock. This trial will generate evidence that may influence current recommendations for MAP targets during management of shock in ICU. The pre-specified protocol summary and statistical analysis plan are presented here.

**Trial registration:**

Prospectively registered on Australian and New Zealand Clinical Trials Registry (ANZCTRN 12623000044628); ClinicalTrials.gov ID NCT05850962 dated 29th April 2023.

**Supplementary Information:**

The online version contains supplementary material available at 10.1186/s13063-025-09142-9.

## Introduction

### Background and rationale

Targeting an adequate mean arterial blood pressure (MAP) with the aid of vasopressors and/or inotropes is one of the fundamental tenets of management of shock in ICU. Although current guidelines strongly recommend a target MAP of 65 mmHg [1–4], it is unclear whether this is the optimal MAP target for each individual patient. Optimal MAP targets may differ among individuals either due to differences in usual pre-illness blood pressure for each individual, different states of tissue autoregulation capacity during critical illness, or other potential unknown reasons for individual variability, possibly including genetic factors [5]. Indeed, blood pressure management to prevent or mitigate new onset organ dysfunction is a priority for future research [6].

In real-world practice, critically ill patients with shock receiving vasopressor or inotrope therapy in ICU are often exposed to a degree of MAP-deficit relative to their usual pre-illness MAP [7]. Recently, we conducted a multicenter prospective cohort study that demonstrated an independent association between time-weighted average MAP-deficit during vasopressor support and subsequent adverse clinical outcomes including 14-day mortality and major adverse kidney events (MAKE-14) [8]. This study highlighted that ICU patients experience near-universal MAP-deficit despite near-perfect maintenance of target MAP of at least 65 mmHg [9]. Additionally, the MAP-deficit was positively associated with 14-day mortality and MAKE-14, suggesting that MAP-deficit could be a more relevant target for haemodynamic management among patients with shock than pursuing a standard MAP threshold of 65 mmHg [3, 4, 8]. Whether minimising such MAP deficit, by adjusting blood pressure targets according to patients’ pre-illness blood pressure (individualised blood pressure management strategy), can improve clinical outcomes remains unknown. On the one hand, individualised MAP target may offer benefit by aligning treatment with the patient’s usual physiological baseline, potentially reducing the risk of organ hypoperfusion, but it may also result in increased vasopressor use. On the other hand, standard MAP target could reduce vasopressor exposure but may also fail to adequately support organ perfusion in patients with higher baseline blood pressure.

Accordingly, we are conducting a multicenter randomised controlled trial (REACT SHOCK RCT; ClinicalTrials.gov ID: NCT05850962) comparing individualised blood pressure target strategy to standard care during vasopressor support among critically ill patients with shock. Our *hypothesis* is that targeting a patient’s pre-illness MAP during management of shock can minimise the degree of MAP-deficit (a measure of relative hypotension), which may help reduce the risk of 14-day mortality and major adverse kidney events in ICU.

### Objectives

The aim of the REACT SHOCK RCT is to determine effectiveness and safety of a strategy, where MAP target during vasopressor therapy for shock in ICU is individualised based on patient’s own pre-illness MAP that would be derived as an average of up to five (and at least two) most recent pre-illness blood pressure readings from within the last 3 years. Our primary objective is to determine whether an individualised MAP target strategy can improve 14-day all-cause mortality compared to standard blood pressure targets among patients with shock. Secondary objectives are to determine whether individualised MAP targets can improve 90-day all-cause mortality, major adverse kidney events by day 14 (MAKE-14), time to death from randomisation to day 14 and day 90, peak increase in serum creatinine, and days alive and free of renal replacement therapy (RRT) by day 28. Safety will be assessed by comparing new adverse events between the two groups.

## Methods: participants, interventions and outcomes

### Trial design and study setting

The REACT SHOCK RCT is an investigator-initiated, pragmatic, international, multicenter, open-label, parallel-group, randomised, standard-care controlled, clinical superiority trial among 1260 critically ill patients, who will be recruited at up to 35 ICUs in Australia, Ireland, Singapore, UK and USA. The trial will be conducted in accordance with Good Clinical Practice guidelines and comply with the principles of the Declaration of Helsinki, national and international regulatory requirements and general data protection regulations. The study is prospectively registered in public registries (ANZCTRN 12623000044628; ClinicalTrials.gov ID NCT05850962) and a pre-specified statistical analysis plan is published here.

### Eligibility criteria

The target population for this trial will comprise ICU patients who satisfy the following criteria and for whom clinicians have an equipoise regarding the target range for optimal blood pressure.

#### Inclusion criteria

- Age ≥ 40 years
- Suspected shock state, defined as clinician-initiated vasopressor/inotropic therapy AND supported by any of the following within the last 24 h:
  - Lactate ≥ 2 mmol/l or base deficit ≥ 3 mmol/l,
  - Urine output ≤ 0.5 ml/kg/h or < 40 ml/h for 2 or more consecutive hours
  - Respiratory rate > 22 per minute
  - Altered mentation (Glasgow Coma Score < 14)

#### Exclusion criteria

- Moribund state or documented not-for-resuscitation orders
- Receiving or imminently needing RRT
- Increase in serum creatinine of more than 350 μmol/l from the baseline level
- End stage renal disease
- At least 24 h have lapsed from the time of initiation of vasopressor or inotropic support
- Trauma is the main reason for the current ICU admission
- Pregnancy, if known
- Active bleeding (clinical suspicion or ≥ 3 packed red blood cells within 24 h)
- Less than two pre-illness blood pressure readings are available from anytime within the last 3 years.
- Patients on extracorporeal support (ECMO, IABP, VAD)
- Potential contraindications to either higher or lower blood pressure targets (including but not limited to)
  - Cerebral perfusion pressure guided therapy (e.g. intracranial haemorrhage or subarachnoid haemorrhage or traumatic brain injury)
  - Abdominal perfusion pressure guided therapy
  - Aortic injury (e.g. dissection or post-operative)
  - Post cardiac surgery
  - Any other condition requiring higher or lower blood pressure target specifically.
- Previously enrolled in REACT SHOCK RCT

### Informed consent

Trained research coordinators or medical doctors at participating ICUs will manually screen patients for eligibility. After determination of eligibility, written informed consent will be obtained from either patients or their legal surrogates.

### Randomisation

Randomisation will be undertaken centrally in a concealed fashion, with a unique computer-generated, site stratified, permuted block randomisation method with random block sizes of 4 and 6. Enrolled patients will be randomly allocated in a 1:1 ratio to either standard care arm or intervention arm.

### Explanation for the choice of comparator

Current guidelines strongly recommend a target mean arterial blood pressure (MAP) of 65 mmHg over higher MAP targets for critically ill patients with shock [1–4]. Therefore, the comparator or the control group will be comprised of patients assigned to standard care that will reflect real-world practice, where vasopressor support is titrated to maintain a default MAP of 65 mmHg unless the treating clinician considers a different MAP target as more appropriate.

### Intervention description

The intervention strategy is to aim for individualised MAP targets that will approximate each individual patient’s usual pre-illness MAP (derived from participant’s own pre-illness MAP based on an average of up to five (at least two) recent pre-illness blood pressure readings from within last 3 years, as validated previously) [8, 10, 11] during vasopressor support in ICU. Pre-illness blood pressure will primarily be obtained from outpatient clinic records, including ambulatory blood pressure monitoring, general practitioner or specialist visits, pre-admission assessments, or echocardiography clinics. Where these records are unavailable, values from the observation charts of a previous hospitalisation within the last 48 h will be used. The pre-illness MAP will be targeted, within the range of 55–95 mmHg, for the duration of vasopressor therapy for up to a maximum of 5 days. The treating clinician can tailor these BP targets if required and as deemed suitable for evolving clinical state. The type of vasopressor that will be used is at the discretion of the treating clinician. If the total additional vasopressor dose required to achieve these individualised MAP targets exceeds 0.75 µg/kg/min (noradrenaline-equivalent [12]), or if in the opinion of the treating clinician the patient is unlikely to benefit or may suffer possible adverse effects, then the blood pressure targets may be adjusted in 5 mmHg steps from the prior level, until a clinically acceptable MAP target is identified.

### Criteria for discontinuing or modifying allocated interventions

Study intervention will cease after 5 days or earlier if a patient is considered well enough by the treating clinician for discharge out of ICU.

### Strategies to improve adherence to interventions

Protocol adherence for enrolled participants will be monitored regularly. Protocol deviation will be defined as failure to adjust dose of vasopressor agents while the MAP remained at least 6 mmHg above or below the set target for 4 consecutive hours, without a documented change of MAP target by the treating clinician. Monitor alarms may be adjusted to prompt appropriate titration of vasopressors or inotropes to maintain target MAP. Study posters or cards (Figure S1) will be provided to participating sites. Cards will show an easy-to-follow algorithm for study-related procedures for each group. Site monitoring will be performed to ensure protocol adherence. The site principal investigator will take primary responsibility for training local staff using study tools provided by the coordinating centre.

### Relevant concomitant care permitted or prohibited during the trial

There are no restrictions to concomitant therapies provided to patients in the study. Participating sites will be requested to adhere to best practice guidelines, regardless of assigned blood pressure targets, in relation to other potentially confounding co-interventions such as fluid management, blood transfusion, sedation interruption, ventilator weaning, nutrition, or use of steroids. Co-enrolment will not be permitted for any other interventional study that is directly related to blood pressure targets.

### Outcomes

The primary outcome is 14-day all-cause mortality. Mortality will be a binary variable, with mortality defined as a record of death within 14 days after randomisation.

Secondary outcomes (Table 3) are:

- 90-day all-cause mortality: All-cause mortality from randomisation to day 90.
- Time to death through day 14: Time to death (hours) will be calculated as the difference between the date and time of death and the date and time of randomisation if death occurs within 14 days of randomisation. Where exact time of death is not available, time will be assumed as 12:00 on the date of death.
- Time to death through day 90: Time to death (days) will be calculated as the difference between the date of death and the date of randomisation.
- Major Adverse Kidney Event (MAKE-14): MAKE-14 is defined as a composite measure of death, new initiation of RRT, or doubling of serum creatinine from the premorbid level at day 14 [13, 14]. Individual components of MAKE-14 will also be reported. The premorbid serum creatinine level will be sourced as the latest available value from medical records within the last year before hospital admission or, if this value was unavailable, as the latest available value during the hospital stay at least 7 days before ICU admission. When neither of these are available, the premorbid serum creatinine will be estimated following the Kidney Disease: Improving Global Outcome (KDIGO) guidelines as described previously [15].
- Renal Replacement therapy (RRT) free days until day 28: Number of days from randomisation to day 28 where the participant is both alive and not dependent on RRT. Patients who die within 28 days will be assigned the worst score of − 1.

### Safety assessment

Adverse events and serious adverse events will be defined according to standard regulatory criteria. Site investigators and research coordinators will prospectively collect safety data through daily review of medical records and direct communication with treating clinicians. The severity, seriousness and causality of each event will be assessed by the local investigator. Prespecified adverse events that will be reported besides death within 28 days of randomisation in hospital are atrial arrhythmia, ventricular arrhythmia, mesenteric ischemia, myocardial ischemia, Takotsubo cardiomyopathy, renal failure requiring RRT, bilateral digital ischemia, cardiac arrest, ischemic stroke and haemorrhagic stroke (Table 4). Additional adverse events defined as potentially or causally related to the study intervention or are otherwise of concern in the judgement of treating clinician will also be recorded. In keeping with established practice of reporting serious adverse events in critical care trials, those events that are deemed part of the natural course of the primary illness or expected complications of the critical illness are not required to be reported [16, 17]. Serious adverse events meeting reporting criteria will be submitted to the coordinating centre within 24 h of site awareness and reported to the relevant human research ethics committees.

### Participant timeline

**Fig. 1:**
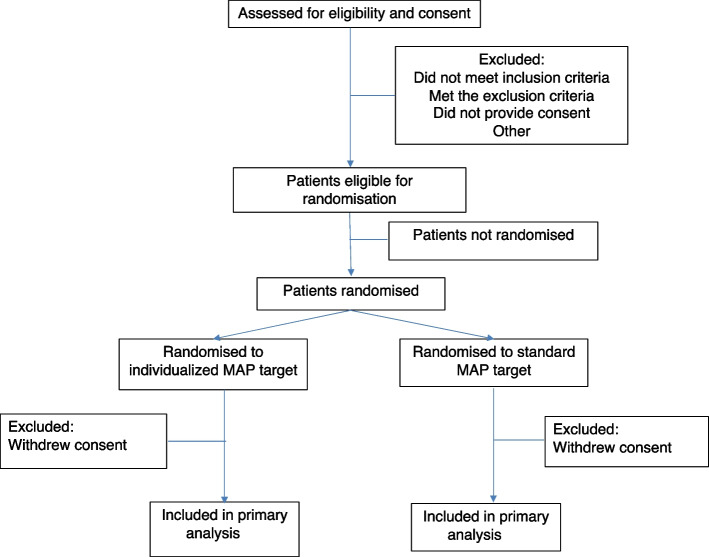
CONSORT flow diagram

A schematic table (Table 1) showing the schedule of enrolment, intervention and assessment in accordance with the SPIRIT guidance [18] is presented. Relevant characteristics of screened patients including date of screening and reason for exclusion will be recorded. Patient flow will be presented as a CONSORT diagram (Fig. 1).

**Table 1:** Schedule of enrolment, intervention and assessments

Activity	Screening	Baseline info	Day 0–5	Day 6–14	Day 28	Day 90
Eligibility criteria evaluation	x					
Informed consent	x					
Demographics	x	x				
Randomisation		x				
Past medical history		x				
Use of nephrotoxic agents		x	x	x		
Vasopressor dose/use		x	x			
Peak serum creatinine		x	x	x	x	
MAP (recorded 4 hourly)		x	x			
Intervention period			x			
Safety outcomes			x	x	x	
Renal replacement therapy			x	x	x	
MAKE-14			x	x		
Mortality			x	x	x	x

### Sample size

Based on our pilot feasibility study [11] and prospective cohort study [8], a sample size of 1260 is required to demonstrate an absolute risk reduction of 6% in the primary endpoint (14-day mortality) in the intervention arm, assuming a 20% incidence of 14-day mortality in the control arm, at an alpha level of 0.05 and power of 80% and allowing for 2.5% attrition. The absolute risk difference of 6% is a conservative estimate relative to the effect size observed in our feasibility study (14-day mortality rate of 13% versus 6% in the standard care versus individualised blood pressure target) [11] or the effect size observed in a pre-specified comparative analysis in our prospective cohort study (14-day mortality rate of 13% versus 22% (*p* = 0.04) in the group with lower-than-median blood pressure deficit versus the group with higher-than-median blood pressure deficit) [8].

### Recruitment

Participating ICUs will have the requisite research infrastructure at each site. We anticipate 30*–*35 ICUs to participate, and based on our previous experience, with a conservative enrolment average rate of 1–2 patients per site per month, the study enrolment would take another 3 years approximately to achieve its sample size. Eligible patients will be identified and screened by the local site research team in consultation with treating clinicians, based on daily review of ICU admissions. Informed consent will be obtained by trained investigators or research coordinators, either directly from patients or, when patients are unable to provide consent, from their legally authorised representatives. To support engagement and motivation across sites, the coordinating centre will conduct regular check-ins with the local research team to discuss site-specific issues and feedback. If recruitment falls behind schedule, contingency plans include additional site training and targeted feedback across participating centres.

### Blinding

Investigators responsible for day-to-day care cannot be blinded due to the nature of the intervention. Analyses will be performed by an independent statistician who will remain blinded to treatment allocation.

### Data collection

Data will be tabulated for patient characteristics (Table 2) and process of care measures (Table 3). This table will include demographic data (age, sex), Acute Physiology and Chronic Health Evaluation (APACHE) III risk score [19], type of shock, comorbidities (chronic hypertension, diabetes mellitus, chronic obstructive pulmonary disease, ischemic heart disease, congestive heart failure, chronic kidney disease, valvular heart disease and peripheral vascular disease), organ system diagnosis (sepsis, gastrointestinal, respiratory, cardiovascular, neurological, renal, haematological, metabolic, musculoskeletal/soft tissue and other), pre-illness blood pressure, period (in weeks) between last pre-illness blood pressure measurement and randomisation, need for mechanical ventilation, volume of intravenous fluid administered within the prior 24 h, fluid balance, exposure to nephrotoxic agents (intravenous contrast, aminoglycoside, vancomycin, non-steroidal anti-inflammatory drug, ACE inhibitor or angiotensin receptor blocker, gancyclovir, calcineurin inhibitor, or remdesivir) within the prior 72 h, pre-morbid creatinine level, MAP at or closest to randomisation, serum lactate and serum creatinine levels obtained at or just prior to time of randomisation, time from ICU admission to randomisation (in hours) and the time from initiation of vasopressor therapy to randomisation (in hours).

**Table 2:** Baseline characteristics

Characteristics	Standard MAP (*n* = xxx)	Individualised MAP (*n* = xxx)
Age, median [IQR], years		
Males, *n* (%)		
APACHE III score, median [IQR]		
Co-morbidities, *n* (%)		
Chronic hypertension		
Diabetes mellitus		
Chronic obstructive pulmonary disease		
Ischemic heart disease		
Congestive heart failure		
Chronic kidney disease		
Valvular heart disease		
Peripheral vascular disease		
Diagnosis organ system, *n* (%)		
Gastrointestinal		
Respiratory		
Cardiovascular		
Sepsis		
Neurological		
Renal		
Metabolic		
Musculoskeletal or soft tissue		
Haematological		
Other		
Type of shock, *n* (%)		
Septic		
Cardiogenic		
Mixed		
Other		
Period between pre-illness blood pressure (BP) measurements and randomisation, median [IQR], weeks		
Number of pre-illness BP readings per patient, *n* (%)		
2		
3		
4		
5		
Pre-illness mean arterial pressure, median [IQR], mmHg		
Receiving invasive mechanical ventilation at randomisation, *n* (%)		
MAP at randomisation, median [IQR], mmHg		
Serum lactate at randomisation, median [IQR], mmol/l		
Pre-illness serum creatinine, median [IQR], micromole/l		
Serum creatinine at randomisation, median [IQR], micromole/l		
Intravenous fluid given within 24 h prior to randomisation, median [IQR], ml		
Fluid balance at randomisation, median [IQR], ml		
Exposure to nephrotoxic agents within 72 h prior to randomisation, *n* (%)		
ACE inhibitor or angiotensin receptor blocker		
Intravenous contrast		
Non-steroidal anti-inflammatory drug		
Aminoglycoside		
Vancomycin		
Calcineurin inhibitor		
Gancyclovir		
Remdesivir		
Number of nephrotoxic agents within 72 h prior to randomisation, median per patient [IQR]		
Time from ICU admission to randomisation, median [IQR], h		
Time from T0^§^ to randomisation, mean (SD), h		

*IQR* interquartile range, *APACHE* Acute Physiology and Chronic Health Evaluation, *BP* blood pressure,

*MAP* mean arterial pressure, *ACE* angiotensin converting enzyme

^§^ T0—Time-point, when vasopressor or inotrope support was initiated

**Table 3:** Exposure variables and other process of care measures

Characteristics	Standard MAP (*n* = xxx)	Individualised MAP (*n* = xxx)
Target MAP on randomisation, median [IQR], mmHg		
MAP-deficit^*^ (time-weighted average), median [IQR], %		
Percentage of time spent with > 20% MAP-deficit^**χ**^, median [IQR], %		
Achieved MAP ^#^ during the treatment period, median [IQR], mmHg		
Time-weighted average vasopressor dose (noradrenaline-equivalent), median [IQR], mcg/kg/min		
Time from randomisation to cessation of vasopressors, median [IQR], h		
Exposure to nephrotoxic agents within 14 days after randomisation, *n* (%)		
ACE inhibitor or Angiotensin receptor blocker		
Intravenous contrast		
Non-steroidal anti-inflammatory drug		
Aminoglycoside		
Vancomycin		
Calcineurin inhibitor		
Gancyclovir		
Remdesivir		
Number of nephrotoxic agents within 14 days after randomisation, median per patient [IQR]		
Exposure to blood transfusion within 5 days after randomisation, *n* (%)		
ICU length of stay post randomisation among survivors, median [IQR], days		
Hospital length of stay post randomisation among survivors, median [IQR], days		

*IQR* interquartile range, *MAP* mean arterial pressure, mmHg

^*^ MAP-deficit = [(pre-illness MAP − achieved MAP)/pre-illness MAP] × 100, using positive incremental area-under-the-curve of MAP-deficit during vasopressor therapy

^**χ**^ % Time spent with > 20% MAP-deficit = [Σ(time-periods with > 20% MAP-deficit)/total time with available MAP data] × 100

^#^ Achieved-MAP was derived as the time-weighted average of 4-hourly values over the active treatment period

**Fig. 2:**
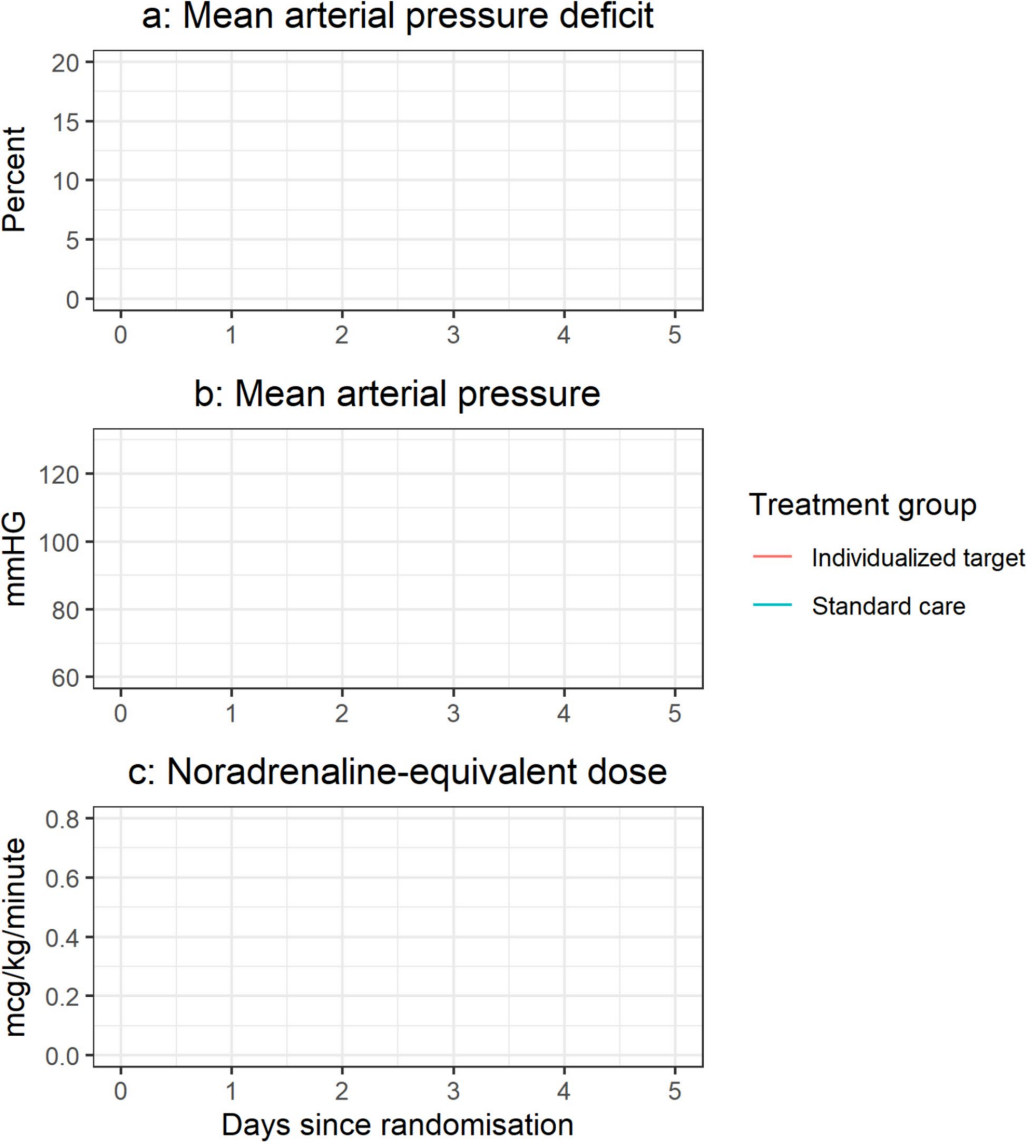
**a–c** Daily means and 95% confidence intervals for time-weighted average percentage MAP deficit, achieved MAP and noradrenaline-equivalent dose

The time when a vasopressor or an inotrope agent is initiated will be identified as T0. Punctual four hourly interval data on MAP and norepinephrine-equivalent vasopressor dose [12] will be monitored until a patient is weaned off vasopressor or inotropic support for at least 24 h or up to a maximum of 5 days, whichever is earlier (Fig. 2). The time-weighted average MAP-deficit, while on vasopressor or inotropic support, will be reported for all patients as a measure of “relative hypotensive load” as described previously [8]. Percentage MAP deficit will be defined as the difference between pre-illness MAP and achieved MAP, divided by pre-illness MAP, expressed as a percentage. A more detailed explanation is provided in the supplement. Pre-illness MAP will be derived as a mean of up to 5 most recent, discrete, pre-illness MAP observations during the 3 years prior to illness, ascertained from past health records. Daily fluid balance and exposure to blood transfusions will also be recorded. Further, exposure to nephrotoxic agents within 14 days of randomisation will be monitored. Safety events such as atrial arrhythmia, ventricular arrhythmia, mesenteric ischemia, myocardial ischemia, Takotsubo cardiomyopathy, bilateral digital ischemia, cardiac arrest, ischemic stroke and haemorrhagic stroke during the study period will be monitored for all patients. Indications to initiate RRT such as presence of metabolic acidosis (pH < 7.2), blood urea nitrogen level of more than 30 mmol/l, hyperkalaemia (K > 6 mmol/l), creatinine level of > 499 micromoles/l, anuria for more than 6 h, fluid overload or other reasons such as toxin removal or clinician’s preference to start early RRT will also be recorded.

### Data management

Data will be electronically entered at each participating site into a REDCap database hosted on Australian-based servers of Hunter Medical Research Institute. Individual access to REDCap database is restricted to study staff only via secure log in with multifactor authentication. Monitoring of the data collection and protocol adherence will be carried out at regular intervals. All the original and updated data-entry will be securely stored to retain an audit trail. At the end of study data collection, integrity checks will be performed to identify any conflicting data, which will be recorded and addressed with a consensus between two study investigators. After all queries are resolved, the database will be locked for final analysis and will be safely stored in a password-protected folder. The database will be securely backed up off site as per standard procedures for data security. Access to the final dataset will be limited to the study statisticians and members of the trial steering group.

## Statistical methods

Analyses will be carried out by a biostatistician using standard statistical software (SAS, Stata and/or R). All randomised patients will be included in an intention-to-treat analysis. Baseline characteristics (Table 2) and other process of care variables (Table 3) will be tabulated for both groups and reported as mean (± standard deviation or median (interquartile range) for continuous variables and as count (%) for categorical variables.

### Primary outcome

Effect estimates for 14-day all-cause mortality will be derived from multivariable logistic mixed effects models with a random intercept for site (assumed normally distributed on the logit scale, with a constant variance) to account for clustering within sites and fixed effects for treatment group, time between T0 (initiation of vasopressor therapy) and randomisation, APACHE III score at randomisation, noradrenaline-equivalent dose at randomisation and type of shock (Table 4). A two-sided *P*-value for the effect of treatment will be obtained from this model and values < 0.05 will be considered statistically significant. The effect estimates of interest will be the relative risk and risk difference (individualised care versus standard care). The relative risk estimate will be obtained as a ratio of the mean for each group of the individual predicted probabilities obtained from the logistic regression model. Differences of these means will be the risk difference. Bootstrapping, using 10,000 iterations, will be used to calculate 95% confidence intervals.

**Table 4:** Outcome measures

	Standard MAP (*n* = xxx)	Individualised MAP (*n* = xxx)	Adjusted^*^ relative risk (95% CI)	Adjusted^*^ risk difference (95% CI)	*P* value
Day 14 all-cause mortality, *n* (%)					
Major Adverse Kidney Event^δ^ within 14 days of randomisation, *n* (%)					
- New requirement for RRT					
- Doubling of creatinine					
RRT- free days^#^ by day 28, *n* (%)					
Day 90 all-cause mortality, *n* (%)					

*RRT* renal replacement therapy

^*^ The model included random intercepts for sites and fixed effects for treatment group, the time between T0 (initiation of vasopressor therapy) and randomisation, APACHE III score at randomisation, vasopressor dose at randomisation and type of shock

^**δ**^ Major adverse kidney event (MAKE)−14 was a composite outcome of death, or new renal replacement therapy during the first 14 days, or doubling of serum creatinine from pre-illness level on day 14 or on day of discharge from ICU, whichever was earlier

^#^ Patients who died were assigned the worst score of − 1

### Secondary outcomes

**Fig. 3:**
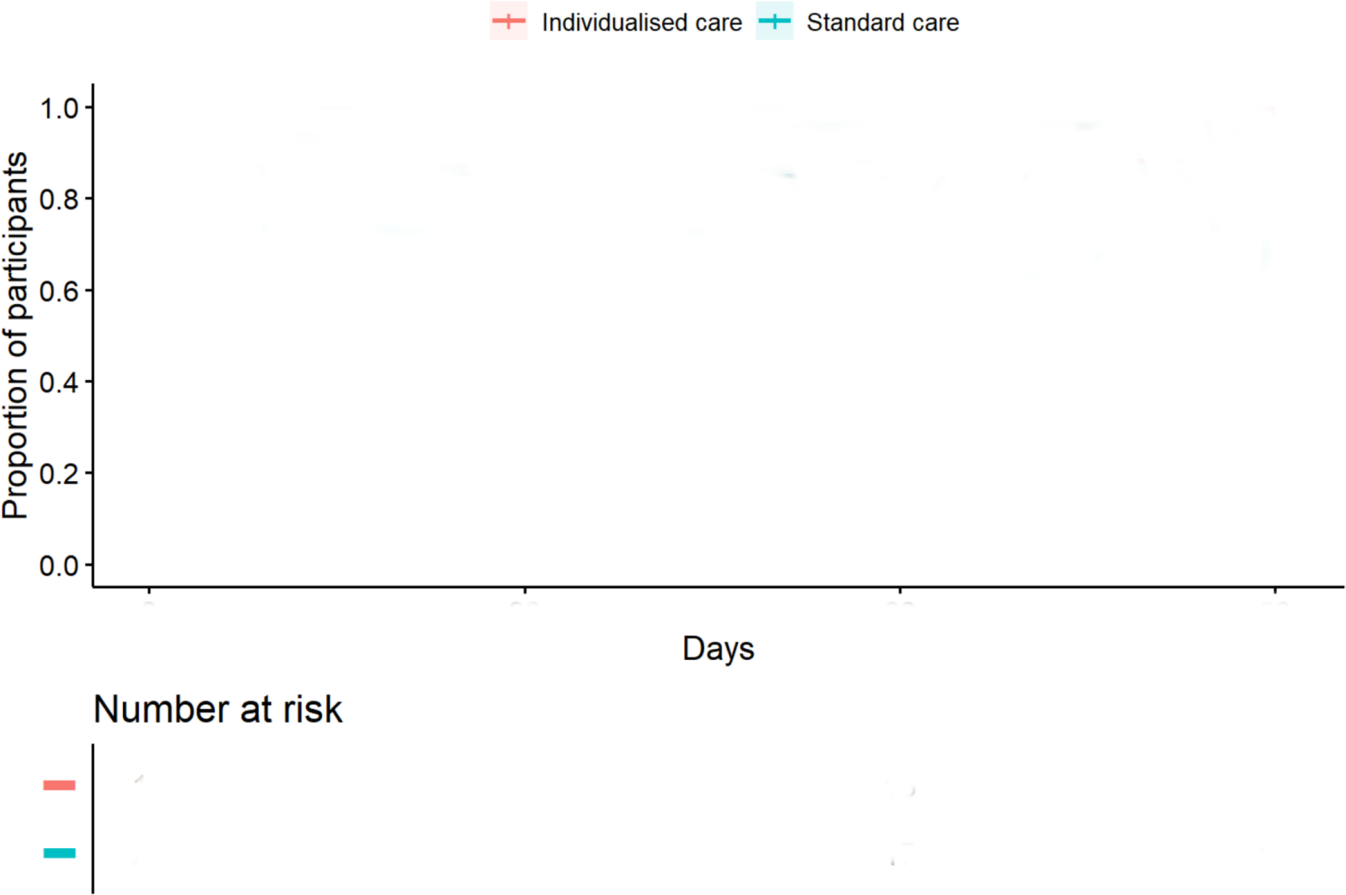
Time-to-event figure for day 14 mortality. The number of people at risk in each group will be listed for days 0, 7 and 14. Cox regression analysis with random effect for site and fixed effects for the exposure adjusted for the time between T0 (initiation of vasopressor therapy) and randomisation, vasopressor dose, APACHE III score at randomisation and type of shock will be used
- RRT-free days by day 28 will be defined as the number of days in the first 28 days post-randomisation when the participant was alive and not dependent on RRT. RRT-free days by day 28 will be analysed as an ordinal outcome, with death assigned the worst value (− 1). Comparisons between groups will be made using a mixed effects proportional odds regression model, with the same fixed and random effects as per the primary outcome model.

- 90-day all-cause mortality and MAKE-14: Analysis will be as described above for 14-day all-cause mortality.
- Time-to-event data will be displayed as Kaplan–Meier curves and log-rank tests will be used to test for significant differences between the two treatment groups (Fig. 3, Figure S6). For time to death, hazard ratios will be estimated with Cox proportional hazards regression, with a random effect for site and fixed effects for the exposure adjusted for the time between T0 (initiation of vasopressor therapy) and randomisation, vasopressor dose, APACHE III score at randomisation and type of shock.

### Safety events

For new adverse events within 28 days, counts and percentages will be reported with *P*-values using Fisher’s exact test where one or more expected count is < 5 or Pearson’s chi-square test, otherwise (Table 5). Win ratio, which is a relatively new approach, appears to be more useful in analysing composite endpoints with varying degree of severity [20] and is increasingly being used as seen in recent RCTs [21, 22]. The win ratio will be calculated by comparing each patient in the treatment group with each patient in the control group to determine who “wins” based on predefined criteria. It provides a hierarchical assessment by first considering the most critical outcomes, such as mortality, before less critical ones, such as adverse events [20]. Hierarchical endpoint in this trial would be a combination of mortality, haemorrhagic stroke, ischemic stroke, cardiac arrest, myocardial ischemia, mesenteric ischemia, renal failure requiring RRT, ventricular arrhythmia, bilateral digital ischemia, takotsubo cardiomyopathy and atrial arrhythmia. Additionally, a sensitivity analysis for the win ratio will be performed using an alternative hierarchy of outcomes using the following order: death, cardiac arrest, ventricular arrhythmia, haemorrhagic stroke, ischaemic stroke, myocardial ischaemia, mesenteric ischaemia, renal failure requiring RRT, bilateral digital ischaemia, Takotsubo cardiomyopathy and atrial arrhythmia. This analysis will assess the robustness of above findings to different event ordering. In addition to win ratio, the net benefit—which is the difference in the proportion of wins between the two groups—will also be reported.

**Table 5:** Adverse events (*N*,%) accrued in hospital within 28 days

	Standard MAP	Individualised MAP	*P* value
Hospital mortality			
Haemorrhagic stroke			
Ischemic stroke			
Cardiac arrest			
Myocardial ischemia			
Mesenteric ischemia			
Renal failure requiring RRT			
Ventricular arrhythmia			
Bilateral digital ischemia			
Takotsubo cardiomyopathy			
Atrial arrhythmia			
Hierarchical composite of above events	Win ratio (95% CI): Net benefit (95% CI):		

*RRT* renal replacement therapy

### Treatment separation between the two arms

A visual summary of MAP-deficit exposure and corresponding vasopressor dose management will be shown for both groups as a stacked bar plot (Figure S2) representing the percentage of time spent in the following mutually exclusive categories: MAP-deficit < 10%; 10–20% with vasopressor dose escalation; 10–20% without vasopressor dose escalation; > 20% with vasopressor dose escalation; > 20 to ≤ 30% without vasopressor dose escalation; and > 30% without vasopressor dose escalation. Differences between the two study arms for the percentage MAP deficit, percentage timepoints spent with more than 20% MAP-deficit, achieved MAP (including highest and lowest daily MAP) (Figure S3, S4) and vasopressor dose during the 5 days following randomisation will be estimated using linear mixed-effects models, with a random intercept for each patient and fixed effects for the treatment group, time since randomisation, noradrenaline-equivalent dose at randomisation, APACHE III score, site and type of shock. All non-missing observations in the period from randomisation to end of 4-hourly follow-up will be used. The effect estimate of interest will be the estimated difference in the marginal means for the two treatment groups from randomisation to day 5, displayed graphically, as well as in a table for 24, 48, 72 and 96 h after randomisation. Achieved MAP and percentage MAP-deficit over time can be presented visually with spaghetti plots with a random sample of patients overlayed with a loess smoother based on all patients.

### Interim analyses

The trial’s steering team will assess accrued adverse events at 10% and 25% of target patient recruitment (126 and 315 patients). The DSMB will review accrued data related to treatment separation (MAP deficit over time) and serious adverse events in both arms after enrolment of 630 participants (50% of the planned sample size). An additional analysis of the primary outcome measure of all-cause 14-day mortality will also be performed during this interim analysis. The study team and the DSMB will remain blinded to the group allocations. Arbitrary dummy group labels, e.g. “A” “B”, will be randomly assigned to the study groups for the interim analysis of the primary outcome variable and different labels, e.g. “G”, “F”, will be assigned to study groups for the analysis of treatment separation. The trial will not be stopped for futility. Following the review of safety events, the DSMB will be required to make a consensus recommendation regarding continuation, modification, or early termination of the trial. In case a consensus decision is unable to be reached, then the DSMB can request an additional interim analysis for further data.

### Subgroup analyses

**Fig. 4:**
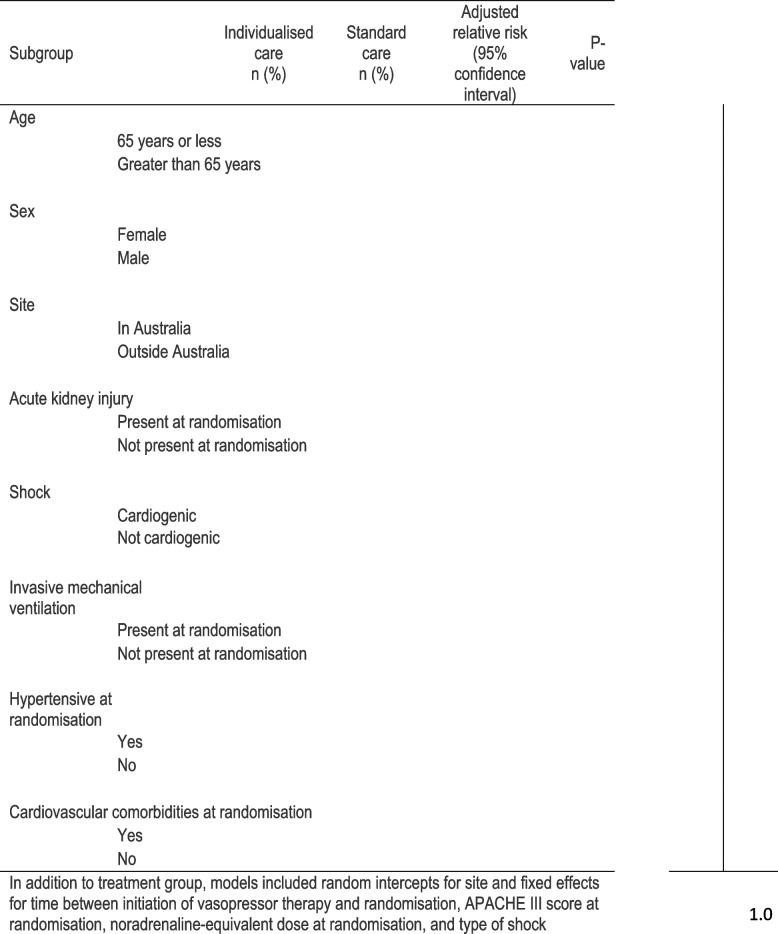
Example of presentation of results for the subgroup analysis for 14-day all-cause mortality as a forest plot

Subgroup analyses for the between-group difference in 14-day mortality, specific to each subgroup, will be conducted (Fig. 4). Pre-specified subgroups will be as follows: age 65 years or less *vs.* greater than 65 years, males *vs.* females, Australian *vs.* non-Australian region, hypertensives vs. non-hypertensives, presence of acute kidney injury *vs.* no acute kidney injury, presence of cardiovascular comorbidities vs. no cardiovascular comorbidities at randomisation, invasive vs. non-invasive mechanical ventilation, and cardiogenic shock *vs.* non-cardiogenic shock at randomisation.

### Missing data or incongruent data

Medical records will be used to ascertain all outcomes. Outliers will be interrogated for validity and impact explored via sensitivity analyses. The intention to treat population for the primary and secondary outcomes is all participants as randomised. It is expected that there will be no, or minimal, missing data for the outcomes. Analysis of outcomes will be complete case analysis and is expected to include the entire intention to treat population or very close to the entire population. MAP and percentage MAP deficit may be missing for some timepoints, but the method of analysis for MAP and percentage MAP deficit does not require all observations to be non-missing. Missing APACHE III score, type of shock and site data is not anticipated. The proportion of data defining the subgroups is also expected to be complete or nearly complete and patients with missing data will not be included in any subgroup analysis. To minimise loss to follow-up, each site will designate a coordinator responsible for outcome tracking, and outcome data will be obtained from hospital records or national mortality registries where required.

### Sensitivity analyses

Impact of any missing outcomes for day 14 and day 90 mortality will be assessed using imputed outcomes under best–worst and worst-best case scenarios. In the best–worst scenario, a “best” outcome event (i.e. did not die) will be assigned in place of all missing outcomes in one treatment arm, and a “worst” outcome event (i.e. died) in place of all missing outcomes in the other treatment arm. The worst-best scenario will be the opposite assignment of outcomes. If the conclusions are similar for these two scenarios, then no missing data analyses will be conducted. However, if conclusions are substantively different, then a multiple imputation analysis will be performed, where missing outcomes will be imputed using chained equations and predictive mean matching, using the five nearest neighbours, in each of the study arms.

Other sensitivity analyses including *per protocol* analysis and *as treated* analysis, based on management of MAP targets during the first 48 h of vasopressor or inotrope therapy post randomisation, will also be conducted. For per protocol analysis, those patients who were not able to be managed as per the specified protocol for the allocated arm will be excluded from each group. For as treated analysis, patients will be grouped into study arms according to the protocol of the study arm that was followed. Determinative thresholds for protocol non-compliance for the control arm will be the mean achieved MAP greater than 75 mmHg and mean MAP-deficit < 8% and mean noradrenaline-equivalent dose > 0.10 mcg/kg/min during the first 24 h of the study period. Whereas determinative thresholds for protocol non-compliance for the intervention arm will be the mean achieved MAP less than 75 mmHg and mean MAP-deficit of 8% or more and mean noradrenaline-equivalent dose < 0.15 mcg/kg/min during the first 24 h of the study period.

### Additional analyses

If the overall intervention effect is significant in the primary analysis, additional exploratory analyses may explore whether MAP-deficit mediates the relationship between the intervention and the primary outcome. A supplementary analysis of 14-day all-cause mortality may also be undertaken within a Bayesian framework (Figure S5), since Bayesian inferences are more intuitive and are helpful in providing more context and there are increasing calls for including Bayesian analysis to complement frequentist analysis for RCTs [23, 24]. This analysis plan is described in detail in the supplement.

### Oversight and monitoring

This RCT will be coordinated by the clinical trials unit at Hunter Medical Research Institute. The trial management group comprises the chief investigator, project manager and clinical trials unit manager, who are responsible for the day-to-day conduct of the trial, including protocol compliance, site coordination and data quality monitoring. The trial steering group comprising of the chief investigator, medical co-investigators and project co-managers oversees trial governance and ensures the study is conducted in accordance with Good Clinical Practice. The trial steering group will conduct regular weekly or bi-weekly meetings. Any protocol variation will be duly approved by relevant regulatory bodies and communicated to all participating sites and collaborators.

### Data safety and monitoring board

The Data Safety Monitoring Board (DSMB) for this trial comprise of three members with scientific expertise in clinical aspects of the patient population being studied, clinical trial conduct, biostatistics and methodology, and who will serve in an individual and independent capacity, free of any professional, intellectual, financial or emotional conflicts of interest in relation to the REACT SHOCK RCT and will provide their expertise and recommendations to the REACT SHOCK investigators. The DSMB members will be objective and unbiased in their assessment of safety and efficacy data provided to them periodically during the conduct of this study.

### Dissemination plans

The trial will be presented at scientific forums and results will be published in peer-reviewed indexed medical journal/s to help disseminate the trial findings to a broad audience.

## Discussion

The aim of the proposed RCT is to determine effectiveness of a strategy, where MAP targets during vasopressor therapy for shock in ICU are individualised based on patients’ own pre-illness MAP. To date no major RCT has tested this strategy among ICU patients with shock. This pivotal trial will provide evidence to fulfil a crucial knowledge gap regarding a common and a fundamental intervention in critical care. This is an opportunity for the clinicians to support a clinical trial that aims to compare the effects of individualised blood pressure targets over standard care for patients with shock in ICU.

The information that will be generated from this research programme has the potential to influence future recommendations regarding haemodynamic management of shock in ICU. In essence, the intervention being tested in this trial is simple to deliver, requires minimal additional clinical support, and if proven effective, will have a potential for significant short-term and long-term benefits for both patients and the health care system. If shown to be beneficial, this intervention can be easily adopted in clinical practice at almost no extra cost to the health care system and has a potential to lead to significant savings. As a pragmatic trial, the range of MAP targets for both arms is well within the realm of real-world practice, while also allowing clinician’s discretion to alter MAP targets if required according to evolving clinical condition for participants.

Overall, there are consistent data from the prior studies in the REACT Shock programme [5, 7, 8, 10, 11] that indicate the need to investigate merits of individualised blood pressure targets in a high-quality RCT. This intervention is directly related to one of the vital clinical parameters and is geared towards reducing potentially unwarranted variation in the degree of blood pressure deficit accepted in standard care.

## Trial status

Current protocol version 4.1 dated 27 March 2024. The trial is actively recruiting and is anticipated to complete recruitment in 2028.

## Sponsor

The University of Newcastle based at University Drive, Callaghan, NSW 2308, Australia. Tel: + 61 2 4921 7307.

## Supplementary Information

Supplementary Material 1.

Supplementary Material 2.

## Data Availability

Not applicable
