# Supplementary material for: REACT SHOCK trial protocol and analysis plan—a multicenter randomised controlled trial comparing individualised blood pressure target versus standard blood pressure target among critically ill patients with shock
Source: Trials. 2025 Oct 14;26:409. doi: 10.1186/s13063-025-09142-9 (PMC12522556; doi:10.1186/s13063-025-09142-9)
Supplement: Supplementary file 1 — Supplementary Material 1. [file 13063_2025_9142_MOESM1_ESM.docx]

**SUPPLEMENT**

**REACT SHOCK trial protocol and analysis plan - A multicenter randomized controlled trial comparing individualized blood pressure target versus standard blood pressure target among critically ill patients with shock.**

**Supplementary content**

- **Bayesian analysis**……………………………………………………………………..……………..………………………………..**3**
- **Figure S1**: Study overview………………………………..……………..……………………………………………………......**5**
- **Figure S2**: MAP-deficit profile in the two groups………………………………………………………………………..**6**
- **Figure S3**: Highest MAP each day..……………..………………………………………………………………………….…..**6**
- **Figure S4**: Lowest MAP each day..……………..…………………………………………………………………….………...**6**
- **Figure S5**: Posterior probability distribution of the relative risk..……………..…………………………….…..**7**
- **Figure S6:** Time-to-event figure for day 90 mortality..………………………....…………….……………….……..**8**
- **Explanation of time-weighted average MAP-deficit**: ..……………..…….………………………………….……..**9**
- **Full list of study investigators and study sites**…………………………………………………………..…..……......**10**
- **References**…………………………..……………..………………………………………………………………………..………….**14**

*Supplementary* *Bayesian analysis*Interest in Bayesian approach is surging particularly for re-analysis of completed trials.^1-4^ Bayesian inferences are more intuitive and there are increasing calls for including Bayesian analysis to complement frequentist analysis for RCTs.^5,6^ Therefore, in addition to the primary (frequentist) analysis, a supplementary analysis of 14-day all-cause mortality may be undertaken within a Bayesian framework. The likelihood function for the number of deaths in each group and a prior distribution that reflects uncertainty in these parameters are specified. The likelihood function and prior distribution will be combined using Bayes’ rule to form a posterior distribution, allowing probabilistic statements to be made.

The effect of treatment group on the likelihood of 14-day all-cause mortality will be estimated with a logistic hierarchical regression model. The model will include a random intercept nested within site (assumed to be normally distributed on the logit scale with a constant variance) and fixed effects for treatment group, APACHE III score at baseline, vasopressor dose at randomisation, and type of shock, as follows:

$$y_{ij}\vee X_{1}\ldots X_{4},\beta_{0}\ldots\beta_{4},V Binomial\left( 1,p_{ij} \right)$$

$ln\left( \frac{p_{ij}}{1-p_{ij}} \right)=\beta_{0}+\beta_{1}X_{1i}+f\left( X_{2i} \right)+\beta_{2}X_{3i}+\beta_{3}X_{4i}+ \beta_{4}X_{5i}+a_{j}$

where:

*i* is the participant

*j* is the site

$X_{1i}$ is the participant’s treatment group

$X_{2i}$is the participant’s baseline APACHE III score

$f(X_{2i})$ is a restricted cubic spline function of $X_{2i}$

$X_{3i}, X_{4i}$ and $X_{5i}$ are indicators of whether the participant had cardiogenic shock, mixed shock and other shock, respectively (septic shock only is the reference category)

$a_{j}$ is the random intercept for site *j* and has a normal distribution with mean 0 and variance V.

The prior distributions for each parameter will be uninformative. The uninformative prior distributions will be:

$\beta_{0},\beta_{1},\beta_{2},\beta_{3},\beta_{4},\beta_{5} Normal\left( 0,1000 \right)$

$$V HalfT_{3}\left( \sigma\right)$$

$\sigma=standard deviation of y$

The relative risk for the individualised treatment group compared to the standard treatment group will be estimated with 95% credible intervals (using Highest Posterior Density intervals) and displayed graphically (Figure S4). The probability that the treatment effect exceeds the minimal clinically important difference (MCID) of an absolute risk reduction in day 14 mortality of 2%, 4% and 6% will also be estimated. These estimates will be obtained using Markov Chain Monte Carlo (MCMC) methods. Specifically, the No-U-Turn Sampler (NUTS) algorithm will be employed to draw samples from the posterior distribution. Convergence of the MCMC chains will be assessed using diagnostic tools such as the Gelman-Rubin statistic (R-hat) and trace plots. Posterior predictive checks will be performed to evaluate the fit of the model to the observed data and ensure the robustness of the inferences.

**Figure S1: Study poster for overview**

**Figure S2: MAP-deficit profile in the two groups**

**Figure S3: Highest MAP each day (mean and 95% confidence intervals) in standard MAP target group versus Individualized MAP target group**

**
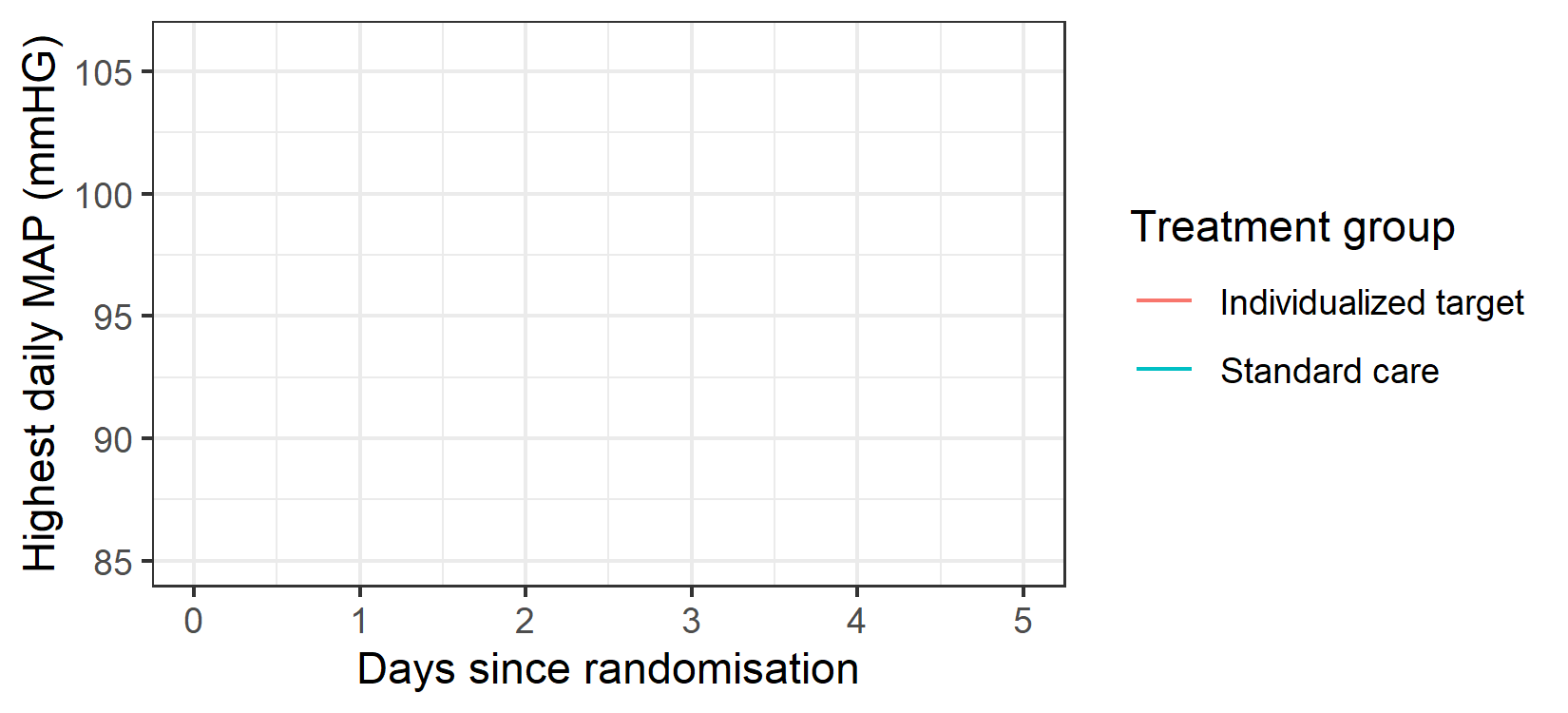
**

**Figure S4: Lowest MAP each day (mean and 95% confidence intervals) in standard MAP target group versus Individualized MAP target group**

**
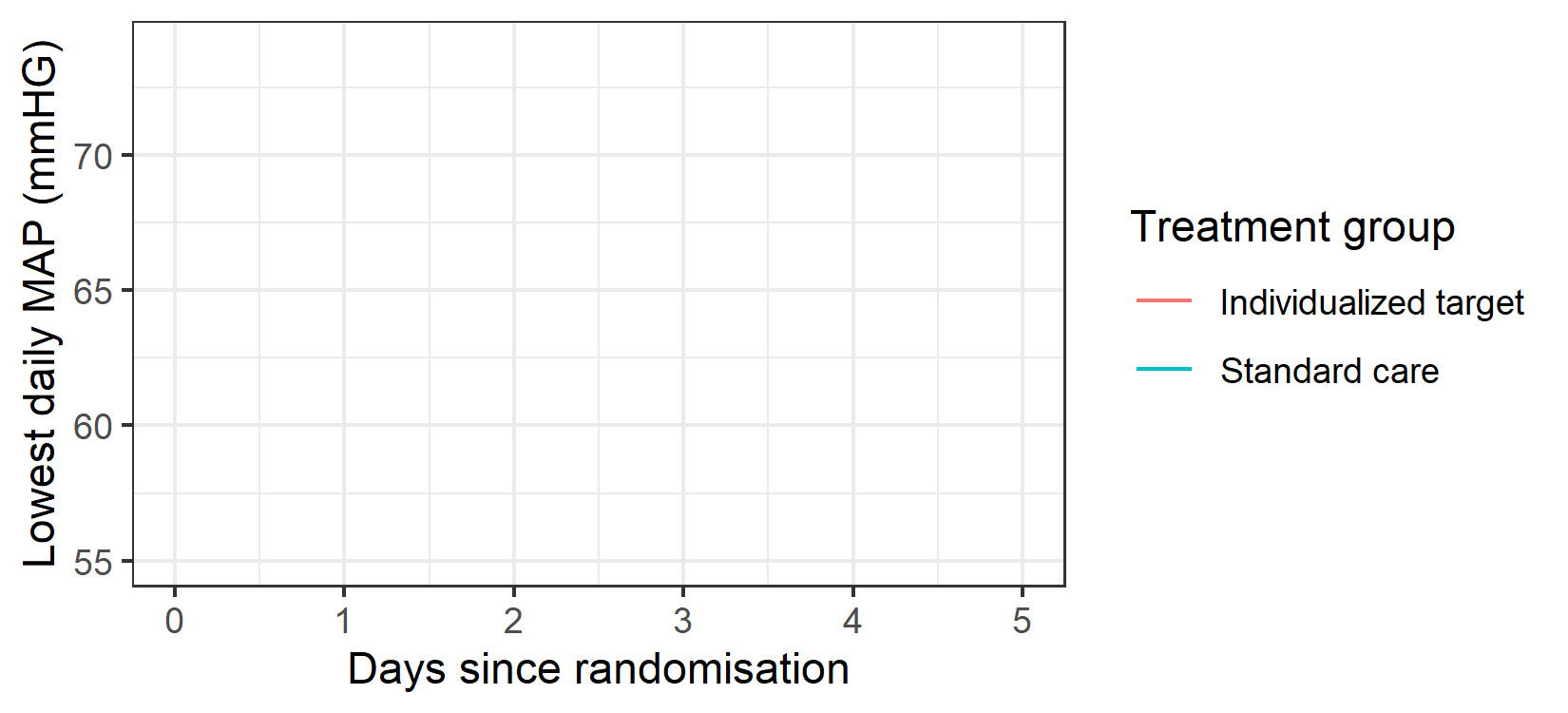
**

**Figure S5: Posterior probability distribution of the relative risk**

**
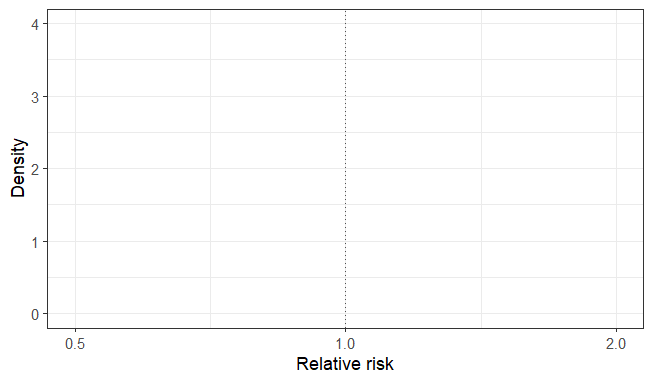
**

**Figure S6:** **Time-to-event figures for day 90 mortality**


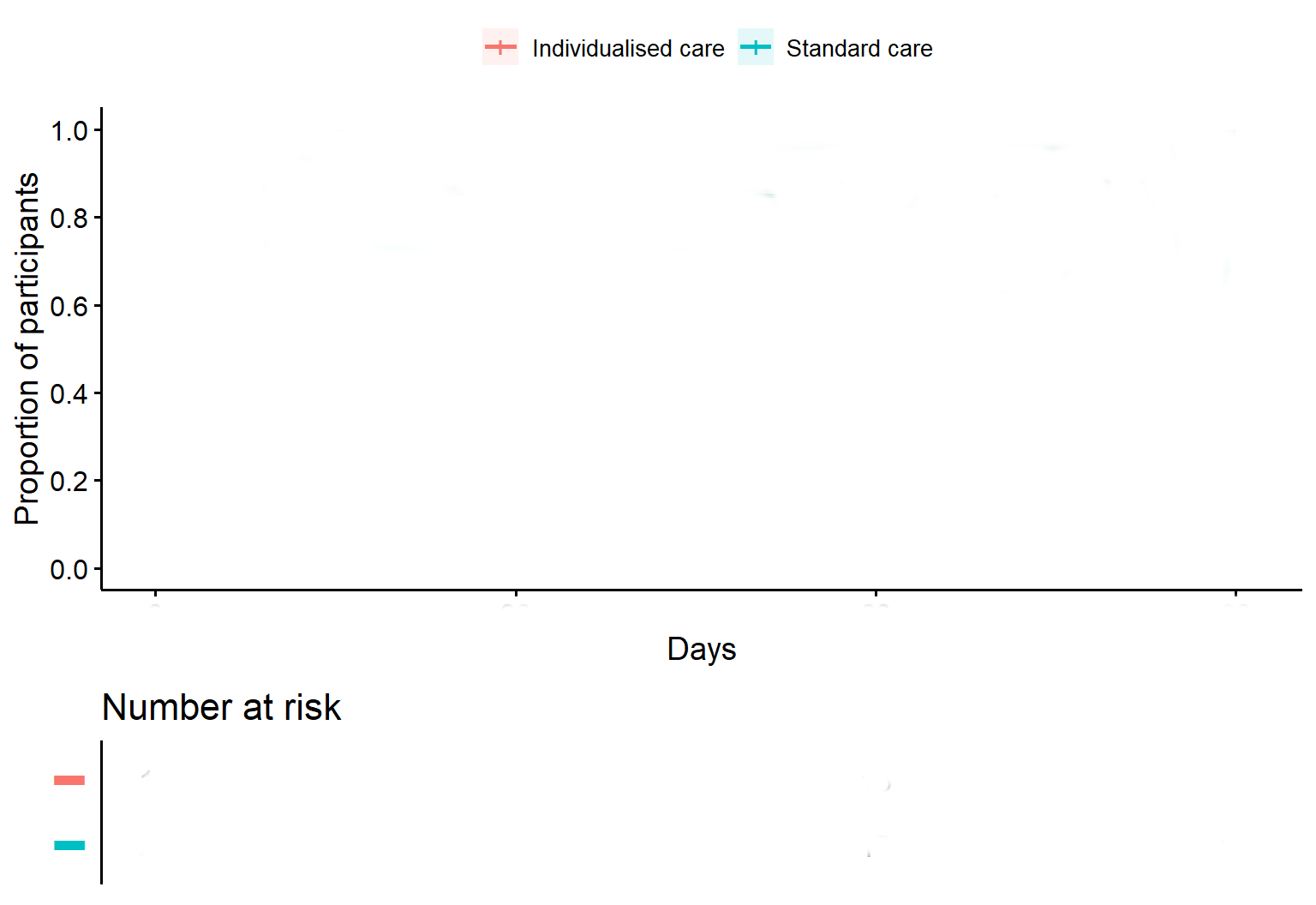


The number of people at risk in each group will be listed for days 0, 30, 60 and 90. Cox regression analysis with random effect for site and fixed effects for the exposure adjusted for the time between T0 (initiation of vasopressor therapy) and randomisation, vasopressor dose, APACHE III score at randomisation and type of shock will be used.

**Explanation of the time-weighted average MAP-deficit**

The ‘time-weighted average MAP-deficit’ is a summary measure of percentage MAP-deficit to which patients are exposed from the time of randomisation to the last recorded MAP during vasopressor therapy in ICU. It is used to quantify the magnitude of average relative hypotension load for each patient (as done previously).^7^ At each four hourly time-interval during the study period, the percentage MAP-deficit will be derived using the equation [% MAP-deficit = 100*[(pre-illness MAP−achieved MAP) /pre-illness MAP], where ‘pre-illness MAP’ will be derived as a mean of patient’s most recent pre-illness blood pressure readings (as explained in the methods) and the ‘achieved MAP’ is the MAP recorded at punctual timepoints on the ICU observation charts. The time-weighted average value for percentage MAP-deficit will then be derived as an aggregate positive incremental area-under-the-curve for % MAP-deficit divided by the cumulative time exposure to vasopressor for each individual patient.^7,8^ An illustration of this concept using hypothetical values is presented below.

**Full list of study investigators and study sites**

|  | Investigators and research co-ordinators | Sites (in alphabetical order) |
| --- | --- | --- |
|  | Naomi Kmetyk – Head of Clinical Trial Operations  Flonda Probert – Project manager | Coordinating site –  Hunter Medical Research Institute (NSW) |
| 1 | Dr. Sayek Khan (PI)  A/Prof Graham Reece (SI)  Treena Sara (SC) | Blacktown Hospital (NSW) |
| 2 | Mahesh Ramanan (PI)  Prashanti Marella (SI)  Emma Williams (SC) | Caboolture Hospital (QLD) |
| 3 | Dr. Vineet Sarode (PI)  Dr. David Brewster (SI)  Dr. Warwick Butt (SI)  Lisa Dougherty (SC) | Cabrini Hospital (VIC) |
| 4 | Dr. Samara Pandham (PI)  Laurence Emery (SC) | Calvary Mater Newcastle (NSW) |
| 5 | Dr. Umesh Kadam (PI)  Sandra Cork (SC)  Patrice Ganuelas (SC)  Seema Radhakrishnan (SC)  Sheenam Soni (SC) | Casey Hospital (VIC) |
| 6 | Raman Azad (PI)  Dr. Nanthini Balakumaran (SI)  Dr. Ramu Adusumalli (SI)  Natalie Luxton  Angela Hammond (SC) | Coffs Harbour Health Campus (NSW) |
| 7 | Dr. Kavitha Shetty (PI)  Dr Narmadhalakshmi Kannabiran (SC)  Dr Kalani Dissanayake (SC) | Fairfield Hospital (NSW) |
| 8 | Dr. Shailesh Bihari (PI)  Shivash Prakash (SI)  Joanne McIntyre (SC)  Julia Brown (SC) | Flinders Medical Centre (SA) |
| 9 | Dr. Ravindranath Tiruvoipati (PI)  Dr Sachin Gupta (SI)  Dr Kavi Haji (SI)  David Zhang (SC) | Frankston Hospital Peninsula Health (VIC) |
| 10 | Dr. Bairbre McNicholas (PI)  Dr. John Laffey (SI)  Dr. John Bates (SI)  Celia Thomas (SC) | Galway University Hospital (IR) |
| 11 | Dr. Atul Gaur (PI)  Eloise Hair (SC)  Shelley Donovan (SC)  Saara Mohamed (SC)  Elisha Turner (SC) | Gosford Hospital (NSW) |
| 12 | Marlies Ostermann (PI)  Jonah Powell-Tuck (SI)  Lais Barroso (SI)  Richelle Santos (SC)  Gillian Radcliffe (SC)  Benjie Cendreda (SC)  Eleonor Hendrie (SC)  Emily Clark (SC)  Fionnuala Walton (SC)  Sarah Fordyce (SC)  Kyma Morera Vas (SC)  Jacinta Obuasi (SC) | Guy’s and St. Thomas’ Hospital (UK) |
| 13 | Dr. Rakshit Panwar (PI)  Dr. Nikhil Kumar (SI)  Dr. Torgeir Westerlund (SI)  Amber-Louise Poulter (SC)  Sarah Dalton (SC) | John Hunter Hospital (NSW) |
| 14 | Dr. Anders Aneman (PI)  Dr. Luis Schulz (AI)  Jennene Miller (SC)  Laurina Luxford (SC) | Liverpool Hospital (NSW) |
| 15 | Dr. Hesham Abdelwahed (PI)  Dr. Vijayalakshmi Venkoba (SI)  Dr. Vinodh Thodur Madapusi (SI)  Merin Jaison (SC) | Maitland Hospital (NSW) |
| 16 | Dr. Alexander Brueton (PI)  Dr Srikanth Tummala (SI)  Dr Sandeep Phaltane (SI)  Dr Gabrielle Beal (SI)  Katherine Plummer (SI)  Ashleigh See (SI)  Lisa Slade (SI)  Jennifer Heath (SI)  Julie Charlton (SC) | Newcastle Private Hospital (NSW) |
| 17 | Dr. Faheem Khan (PI)  Dr Amit Kansal (SI)  Dr Monica Gulati Kansal (SI)  Manasa Sachin Kumar (SC) | Ng Teng Fong General Hospital (SG) |
| 18 | Dr. Ravindranath Tiruvoipati (PI)  Hannah Rule (SC)  David McArdle (SC) | Peninsula Private Hospital (VIC) |
| 19 | Dr. Craig Hore (PI)  Dr. Ruth Herod (SI)  Dr. Rob Hislop (SI)  Ann Bodill (SC)  Tonia Woodberry (SC) | Port Macquarie Base Hospital (NSW) |
| 20 | Dr. Jayesh Dhanani (PI)  Dr. Dougal Carlisle (SI)  Janine Stuart (SC)  Melissa Lassig-Smith (SC)  Amelia Livermore (SC) | Royal Brisbane & Women's Hospital (QLD) |
| 21 | Dr. Anthony Delaney (PI)  Dr. Lachlan Donaldson (SI)  Dr. Christopher Andersen (SI)  Elizabeth Yarad (SC)  Rosalind Elliott (SC)  Frances Bass (SC)  Tessa Garside (SC)  Anne OConnor (SC) | Royal North Shore Hospital (NSW) |
| 22 | Dr. Frank van Haren (PI)  Dr. Manoj Saxena (SI)  Dr. Kush Deshpande (SI)  Chloe Edwards (SC)  Sarah Valle (SC)  Rebecca Sidoli (SC) | St George Hospital (NSW) |
| 23 | Dr. Humphrey Walker (PI)  Dr. Alastair Brown (SI)  Jennifer Holmes (SC)  Melissa King (SC)  Viean Luk (SC)  Adelle Odering (SC) | St Vincent’s Hospital Melbourne (VIC) |
| 24 | Dr. Alistair Nichol (PI)  Kathy Brickell (SC)  Marina Sweeney (SC) | St Vincent's University Hospital, Dublin (IR) |
| 25 | Dr. Anas Naeem (PI)  Elizabeth Turner(SC)  Kelsey Matthews (SC) | Sutherland Hospital (NSW) |
| 26 | Dr. Yvelynne Kelly (PI)  Dr. Thomas Gordan (SI)  Dr. Muhammad Tahir (SI)  Dr. Abubakar Obaid (SI)  Dr. Maeve Egan (SI)  Sabina Mason (SC)  Rachael Gitau (SC) | Tallaght University Hospital (IR) |
| 27 | Dr. Sonia Langlais (PI)  Dr Marie Scott (SI)  Kalai Kanagasingham (SI)  Valinda Wesble (SC)  Jazmin Moore (SC) | Tamworth Hospital (NSW) |
| 28 | Dr. Yew Woon Chia (PI)  Dr. Joo Hor Tan (SI)  Jia Min Kang (SC)  Dylan Lim (SC) | Tan Tock Seng Hospital (SG) |
| 29 | Dr. Sananta Dash (PI)  Karen Carson (SC)  Monica Watson (SC) | Townsville University Hospital (QLD) |
| 30 | Dr. Joao Pedro Teixeira (PI)  Dr Nathan Nielsen (SI)  Dr Seth Skiles (SI)  Dr David Jackson (SI)  Natalie Weiss (SC)  Kenda Deputy (SC)  Liam Goodale (SC)  Valeria Mejia (SC) | University of New Mexico (USA) |
| 31 | Dr. Ashish Khanna (PI)  Dr Ryan Maves (SI)  Dr Bryan Marchant (SI)  Dr Megan Hicks (SI)  Dr Casey Bryant (SI)  Dr Karuna Rajkumar (SI)  Lynnette Harris (SC)  Tanmay Sura (SC)  Jessica Reeves (SC)  Brandon Reeves (SC)  Amelia Eaton (SC) | Wake Forest School of Medicine (USA) |
| 32 | Dr. Matthew MacPartlin (PI)  Wenli Geng (SC) | Wollongong Hospital (NSW) |

**References**

1. Goligher EC, Tomlinson G, Hajage D, et al. Extracorporeal Membrane Oxygenation for Severe Acute Respiratory Distress Syndrome and Posterior Probability of Mortality Benefit in a Post Hoc Bayesian Analysis of a Randomized Clinical Trial. *JAMA*. Dec 04 2018;320(21):2251-2259. doi:10.1001/jama.2018.14276

2. Zampieri FG, Machado FR, Veiga VC, et al. Determinants of fluid use and the association between volume of fluid used and effect of balanced solutions on mortality in critically ill patients: a secondary analysis of the BaSICS trial. *Intensive Care Med*. Jan 2024;50(1):79-89. doi:10.1007/s00134-023-07264-9

3. Zampieri FG, da Costa BR, Vaara ST, et al. A Bayesian reanalysis of the Standard versus Accelerated Initiation of Renal-Replacement Therapy in Acute Kidney Injury (STARRT-AKI) trial. *Crit Care*. Aug 25 2022;26(1):255. doi:10.1186/s13054-022-04120-y

4. Zampieri FG, Damiani LP, Bakker J, et al. Effects of a Resuscitation Strategy Targeting Peripheral Perfusion Status versus Serum Lactate Levels among Patients with Septic Shock. A Bayesian Reanalysis of the ANDROMEDA-SHOCK Trial. *Am J Respir Crit Care Med*. Feb 15 2020;201(4):423-429. doi:10.1164/rccm.201905-0968OC

5. Wijeysundera DN, Austin PC, Hux JE, Beattie WS, Laupacis A. Bayesian statistical inference enhances the interpretation of contemporary randomized controlled trials. *J Clin Epidemiol*. Jan 2009;62(1):13-21.e5. doi:10.1016/j.jclinepi.2008.07.006

6. Zampieri FG, Casey JD, Shankar-Hari M, Harrell FE, Harhay MO. Using Bayesian Methods to Augment the Interpretation of Critical Care Trials. An Overview of Theory and Example Reanalysis of the Alveolar Recruitment for Acute Respiratory Distress Syndrome Trial. *Am J Respir Crit Care Med*. Mar 01 2021;203(5):543-552. doi:10.1164/rccm.202006-2381CP

7. Panwar R, Tarvade S, Lanyon N, et al. Relative Hypotension and Adverse Kidney-related Outcomes among Critically Ill Patients with Shock. A Multicenter, Prospective Cohort Study. *Am J Respir Crit Care Med*. 11 15 2020;202(10):1407-1418. doi:10.1164/rccm.201912-2316OC

8. Panwar R, Van Haren F, Cazzola F, Nourse M, Brinkerhoff G, Quail A. Standard care versus individualized blood pressure targets among critically ill patients with shock: A multicenter feasibility and preliminary efficacy study. *J Crit Care*. May 04 2022;70:154052. doi:10.1016/j.jcrc.2022.154052
